# Human Metapneumovirus Infection among Children Hospitalized with Acute Respiratory Illness

**DOI:** 10.3201/eid1004.030555

**Published:** 2004-04

**Authors:** James A. Mullins, Dean D. Erdman, Geoffrey A. Weinberg, Kathryn Edwards, Caroline B. Hall, Frances J. Walker, Marika Iwane, Larry J. Anderson

**Affiliations:** * Centers for Disease Control and Prevention, Atlanta, Georgia, USA; † University of Rochester School of Medicine & Dentistry, Rochester, New York, USA; ‡ Vanderbilt University, Nashville, Tennessee, USA

**Keywords:** Respiratory tract infections, metapneumovirus, Respiratory Syncytial Virus, prospective studies

## Abstract

Recent studies have associated human Metapneumovirus (HMPV) infection in children with respiratory disease of similar severity as respiratory syncytial virus (RSV) infection. We studied 668 banked swab specimens (one per admission) collected from a population-based, prospective study of acute respiratory illness among inpatient children from two U.S. cities. Specimens were tested for HMPV, RSV, influenza, and parainfluenza viruses by reverse transcription–polymerase chain reaction assays. Twenty-six (3.9%) were positive for HMPV; 125 (18.7%) for RSV; 45 (6.7%) for parainfluenza 1, 2, or 3; and 23 (3.4%) for influenza. HMPV-positive children were significantly older than RSV-positive children. HMPV-positive children required medical intensive care and received supplemental oxygen in similar frequencies to RSV-positive children. Among children hospitalized with respiratory illness, the incidence of HMPV infection was less than RSV, but clinical disease severity mirrored that of RSV infection. Further investigations to better characterize HMPV infection and its clinical effect are needed.

Human Metapneumovirus (HMPV) is a recently discovered respiratory pathogen of the family *Paramyxoviridae* belonging to the same subfamily, *Pneumovirinae*, as respiratory syncytial virus (RSV) (*1*,*2*). HMPV was first recognized in the Netherlands in 2001 in nasopharyngeal aspirate samples collected from children over a 20-year period (*1*). It has since been identified in Canada (*3*,*4*), Australia (*5*), the United Kingdom (*6*), France (*7*), Hong Kong (*8*) and the United States (*9*). Recent reports investigating HMPV have shown that HMPV-infected children who are hospitalized with respiratory illness frequently have clinical diagnoses of bronchiolitis and pneumonia, much like children infected with RSV (*1*,*4*,*5*,*7*,*10*). Clinical symptoms from HMPV-infected children have included nonproductive cough, nasal congestion, and wheezing (*5*,*6*,*8*,*11*). The most commonly reported abnormality on chest radiography was bilateral infiltrates, indicative of pneumonia (*5*,*11*). One child with influenzalike illness seen in an outpatient clinic was reported to have HMPV and negative cultures for influenza virus and RSV (*6*).

A recent study in Hong Kong looked at HMPV infection by using a prospective, population-based design (*8*), but no such studies on HMPV infection have been reported that used U.S. data. We present results of such studies and confirm the effect of HMPV infections on acute respiratory illness (ARI) hospitalizations in children.

## Methods

### Study Design

The design of the parent study has been described previously (*12*). The present study was conducted as part of the New Vaccine Surveillance Network, a population-based active surveillance network for vaccine-preventable diseases related to new vaccines. The study samples were collected at two hospitals in Rochester, New York and three hospitals in Nashville, Tennessee; each cares for >95% of children hospitalized in their respective counties (Monroe County, New York and Davidson County, Tennessee). The children from these two counties provide population-based data. Rochester hospitals also contributed samples from children who resided in six outlying counties. The data from the outlying counties are not regarded as population-based since those counties are also served by their outlying hospitals. Samples were collected from August 2000 through September 2001 from children <5 years of age hospitalized for ARI or ARI-related diagnoses.

Combined nose and throat swab specimens from each child were cultured at the study sites for influenza virus A and B; RSV; and parainfluenza viruses 1, 2, and 3. HMPV had not yet been discovered at the beginning of the trial and, therefore, was not investigated. Frozen aliquots of each specimen were analyzed for the above viruses plus HMPV by using reverse transcription–polymerase chain reaction (RT-PCR) assays and GeneScan deoxyribonucleic acid fragment analysis of PCR products. All viruses were confirmed from a second aliquot of sample. A positive virus culture or confirmed RT-PCR was considered a positive test for respiratory viruses other than HMPV.

### RT-PCR Process for HMPV

The RT-PCR assays for HMPV were performed as described previously (*13*). Briefly, RNA extracts were prepared from 100 μL of specimen by using the automated NucliSens extraction system (bioMérieux, Durham, NC). Oligonucleotide primers used for RT-PCR were designed to conserved regions of the HMPV nucleoprotein and fusion protein genes, and one primer for each set was 5′-end-labeled with fluorescein (6-FAM) to facilitate GeneScan analysis. One-step amplification reactions were performed by using the Access RT-PCR System (Promega Corp., Madison, WI). HMPV-positive and -negative controls containing a standardized viral RNA extract and nuclease-free water, respectively, were included in each assay. Amplification products were analyzed on an ABI Prism 310 Genetic Analyzer (Applied Biosystems, Foster City, CA) with GeneScan software (ver. 3.1.2). Electropherograms were reviewed, and amplification products identified within 2 nt of the expected size (195 nt for nucleoprotein gene; 347 nt for the fusion protein gene) were considered “preliminary” positives. Preliminary positives were then repeat-tested with a new extraction, when an additional specimen aliquot was available. Because viral culture for HMPV was unavailable, specimens were designated “true” positives if the same amplification product for both genes was identified on repeat testing of the sample extract.

### Statistical Analysis

Epidemiologic data were analyzed by using Statistical Analysis Software (SAS) version 8.0 (SAS Institute) and EpiInfo 6.04d (Centers for Disease Control and Prevention, Atlanta, Georgia). Statistical significance was determined by using the chi-square test with α= 0.05. We include data from the eight counties in all analyses in this paper except for the population-based incidence rates, which include Monroe and Davidson County data only, the principal counties served by the study hospitals. Population-based annual incidence rates per 100,000 children were calculated for a 12-month period, October 2000 through September 2001, by weighting the observed number of enrolled, hospitalized cases to account for sampling days per week and nonresponse by age, dividing by the 2000 U.S. Census population count, and multiplying by 100,000. Ninety-five percent confidence intervals (CIs) were calculated by bootstrap methods (*14*)

## Results

Six hundred sixty-eight samples were analyzed from 641 children enrolled in the network from August 2000 through September 2001 (27 children were hospitalized twice and eligible to participate both times). Enrollment by site was evenly distributed (Table 1), and no differences in age or sex distributions of the participants were noted between the sites. However, the Rochester site enrolled significantly more white non-Hispanic children in the study than the Nashville site (risk ratio [RR] = 1.3, p = 0.0002), representing differences in overall racial distribution between the sites. More male patients participated in the study than female patients, reflecting the higher proportion of ARI hospitalizations for boys (57%), and the median age of study participants was 6 months (range <1–59 months). Among the 668 samples, 26 (3.9%) tested positive for HMPV; 125 (18.7%) tested positive for RSV; 45 (6.7%) tested positive for parainfluenza viruses 1, 2, or 3; and 23 (3.4%) tested positive for influenza virus.

**Table 1:** Characteristics of HMPV-positive patients, RSV-positive patients, and the rest of the study cohort, New Vaccine Surveillance Network acute respiratory illness study, August 2000–September 2001

Characteristic	HMPV-positive, n = 26 (%)	RSV-positive, n = 125 (%)	Rest of cohort, n = 517 (%)
Median age	11.5 mo^a,c^	3 mo	7 mo
Range	1–43 mo	<1–56 mo	<1–59 mo
Age distribution			
<6 mo	5 (19)^b,c^	73 (58)^a,c^	244 (47)
6 mo–1 y	8 (31)^b,c^	26 (21)^a,c^	80 (15)
1–2 y	11 (42)^b,c^	19 (15)^a,c^	82 (16)
>2 y	2 ( 8)^b,c^	7 ( 6)^a,c^	111 (22)
Male	18 (69)	72 (58)	295 (57)
Race			
White (non-Hispanic)	17 (65)	75 (60)	256 (50)
Black (non-Hispanic)	8 (31)	32 (26)	172 (33)
Hispanic	1 ( 4)	13 (10)	55 (11)
Other	0 ( 0)	5 ( 4)	26 ( 5)
Unknown	0 ( 0)	0 ( 0)	8 ( 2)
Underlying medical condition	14 (54)^a,d^	36 (29)	163 (31)^b,d^
Born premature	8 (31)	19 (16)	77 (15)
Breastfed	16 (62)	71 (58)	274 (53)
Exposed to household smoker	13 (50)	50 (41)	236 (46)
Attended daycare	8 (31)	31 (25)	167 (32)
Site			
Nashville	9 (35)	59 (47)	282 (55)
Rochester	17 (65)	66 (53)	235 (45)

^a^Comparison of HMPV-positive to RSV-positive patients. HMPV, human metapneumovirus; RSV, respiratory syncytial virus. ^b^Comparison of HMPV-positive patients to rest of cohort. ^c^p<0.001. ^d^p<0.05.

### HMPV Patient Description

The remaining analyses in this section describe the 26 HMPV-positive patients and compare them with the 125 RSV-positive patients and the rest of the cohort (517 patients who were neither HMPV- nor RSV-positive). The 26 HMPV-positive patients were mostly male (18 [69%]), and 17 (65%) were from the Rochester study site. The median age of HMPV-positive patients was significantly higher than that of the RSV-positive patients (11.5 months and 3 months, respectively; p < 0.0001) and approached significance when compared to the median age of the rest of the cohort (11.5 months and 7 months, respectively; p = 0.06). Most of the HMPV-positive patients were 6 months to 2 years of age compared with an age of <6 months for most of the RSV-positive patients (p = 0.0015) and the rest of the cohort (p = 0.0002) (Table 1).

Among the 26 HMPV-positive children, 8 (31%) were reported to have been born >1 month premature, and 16 (62%) were breastfed. Half of the HMPV-positive children were exposed to a household smoker, and eight (31%) attended daycare >4 hours per week. These findings were not significantly different from those for the RSV-positive children or the rest of the cohort (Table 1).

Preexisting medical conditions were assessed both by parent or guardian interviews and medical record reviews. Fourteen (54%) HMPV-positive children had serious underlying medical conditions, compared with 36 (29%) of RSV-positive children (RR = 2.4, p = 0.01) and 163 (32%) of the rest of the cohort (RR = 2.4, p = 0.02) (Table 1). Among the 14 HMPV-positive children with underlying conditions, the most frequently reported conditions were asthma (8 patients) and heart disease (3 patients). Three children had more than one condition reported.

### HMPV Clinical Description

Admission diagnoses were captured for all patients enrolled in the study. Pneumonia (6 patients), bronchiolitis (6 patients), asthma (3 patients), and fever without localizing signs (3 patients) were the top four admission diagnoses mentioned for HMPV-positive patients. For RSV-positive patients, bronchiolitis (54 patients), RSV (47 patients), pneumonia (33 patients), and asthma (20 patients) were the admission diagnoses most frequently mentioned.

Among the 26 HMPV-positive patients, 24 (92%) had cough as a symptom. This finding was similar to the percentage of RSV-positive patients, but it was a significantly greater percentage than the 64% of the cohort who experienced cough (RR = 1.7, p = 0.0001). Other predominant symptoms of HMPV-positive patients included 19 (73%) with fever, 20 (77%) with nasal congestion, and 21 (81%) with shortness of breath. During the course of their hospital stays, HMPV-positive patients tended to be admitted to intensive care units more than the rest of the cohort (RR = 2.8, p = 0.06), and they were more likely to receive supplemental oxygen while hospitalized (RR = 1.8, p = 0.008) (Table 2).

**Table 2:** Clinical characteristics of HMPV-positive patients compared with RSV-positive patients and the rest of the study cohort, New Vaccine Surveillance Network acute respiratory illness study, August 2000–September 2001

	HMPV-positive, n = 26	RSV-positive, n = 125	Rest of cohort, n = 517
Characteristic	n (%)	n (%)	n (%)
Presenting Symptoms			
Cough	24 (92)^a,b^	124 (99)	333 (64)
Fever	19 (73)	85 (68)	383 (74)
Nasal Congestion	20 (77)	110 (88)	329 (64)
Shortness of Breath	21 (81)	119 (95)	334 (65)
Hospital Course			
Admitted to ICU	4 (15)	6 ( 5)	28 ( 5)
Received supplemental O_2_	14 (54)^a,c^	76(61)	51 (29)
Intubated	2 ( 8)	3 (2)	10 (2)
Chest x-ray findings	(n = 23)	(n = 105)	(n = 304)
Chest x-ray performed	23 (88)^a,c^	105 (84)	304 (59)
Any pneumonia/infiltration	9 (39)	25 (23)	77 (25)
Hyperinflation	11 (48)	54 (51)	103 (34)

^a^Comparison of HMPV-positive patients to rest of cohort. ^b^p<0.001. ^c^p<0.01

Twenty-three (88%) HMPV-positive patients received chest radiographs during their hospital stay. This percentage was similar to 84% of RSV-positive patients who received chest radiographs but significantly more than the rest of the cohort, of whom only 59% received chest radiographs (RR = 1.5, p = 0.005). Nine (39%) HMPV-positive patients had radiologic evidence of pneumonia or pulmonary infiltration, while 11 (48%) had evidence of hyperinflation. These percentages were not significantly different from the RSV-positive patients and the rest of the cohort (Table 2).

Up to 10 discharge diagnoses were recorded for each study patient. The most frequently mentioned clinical diagnoses among all discharge codes for HMPV-positive patients were asthma (7 patients), pneumonia (6 patients), bronchiolitis (6 patients), hypovolemia (5 patients), and otitis media (4 patients). These discharges were similar to those coded for RSV-positive patients.

Seasonality for HMPV was estimated by using admission dates for HMPV-positive children. The earliest case appeared in Rochester on January 1, 2001. The first case in Nashville appeared 7 weeks later on February 19, 2001. Though most HMPV cases occurred from January to April 2001, Nashville identified one case each in May and in August (Figure).

**Figure:**
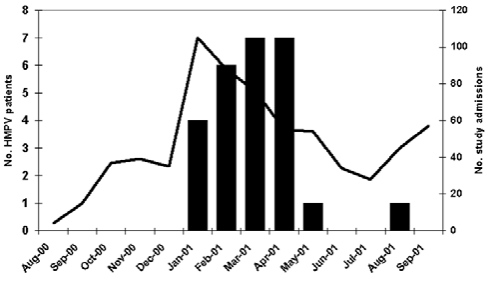
Distribution of HMPV patients and overall study admissions by month of admission, New Vaccine Surveillance Network acute respiratory illness study, Aug 2000–Sept 2001. Black bars represent HMPV-positive patients, while the line represents study admissions.

### HMPV Population-based Hospitalization Rates

To estimate population-based hospitalization rates, we focused only on cases that resided in Davidson and Monroe Counties. Of the 26 HMPV-positive cases, 19 (73%) resided in these two counties, 9 in Davidson County and 10 in Monroe County. Adjusted for age, the estimated hospitalization rates per 100,000 persons for HMPV infection were 114 cases (95% CI 47 to 198) for children <12 months of age, 131 cases (95% CI 51 to 225) for children 1–2 years of age, and 10 cases (95% CI 0 to 20) for children 2–5 years of age.

## Discussion

These data suggest that among children <5 years of age hospitalized with respiratory illness, HMPV is associated with a rate of community-acquired ARI similar to that of the combined parainfluenza viruses 1–3 and influenza viruses but substantially less than that associated with RSV. The incidence of 3.9% of positive HMPV specimens among those tested is slightly greater than the 2.3% reported in one Canadian study (*10*) and slightly less than the 5.5% reported in a Hong Kong study (*8*) of similar design. Other studies have found that HMPV incidence can vary from year to year, sometimes rivaling or exceeding RSV incidence (*13*,*15*). Our single year of data did not allow us to evaluate this.

As reported in other HMPV studies, clinical symptoms of HMPV-positive patients mirrored those of RSV-positive patients. While this comparison could indicate that HMPV may be associated with a similarly severe respiratory illness such as RSV, the clinical care information is more revealing. The tendency for HMPV-positive patients to be admitted to the intensive care unit and their increased requirement for supplemental oxygen during their hospital stay occurred with similar frequency to RSV-positive patients (Table 2). Therefore, while the incidence of HMPV infection may not compare with that of RSV infection, the disease severity may be very similar.

The observed illness severity could have been influenced by underlying medical conditions reported in half of HMPV-positive patients. Indeed, three of four HMPV-positive children admitted to the ICU and 8 of 14 requiring supplemental oxygen had underlying medical conditions. However, proportions of underlying medical conditions were not significantly different for RSV-positive patients or the rest of the cohort that required these same hospital services.

Asthma was the most frequently recorded discharge diagnosis among HMPV-positive children. A review of individual cases showed that six of the seven HMPV-positive children with asthma as a discharge diagnosis also had asthma as an underlying medical condition. Two of these children were specifically diagnosed with acute exacerbation of asthma. This finding is similar to findings from a recent HMPV study in Finland that suggested HMPV may stimulate asthmatic episodes in children with asthma (*7*), but it contrasts findings from an Australian study that showed no such association (*16*).

Our study found that HMPV-infected children were significantly older (median age 11.5 months) than RSV-positive children (median age 3 months) (Table 1). This age difference was also noted in the Hong Kong study (*8*), and our median age for RSV-positive patients is consistent with previous reports (*17*). With other clinical aspects of HMPV infection so similar to RSV, one might have expected the age distribution of HMPV-positive patients to be similar as well. The difference could be due to longer lasting maternal immunity to HMPV compared with RSV, or perhaps the pathogenesis of HMPV disease favors older children. More research is needed to answer these questions.

Our year-round surveillance for respiratory illness showed that HMPV infection tended to occur mainly during the winter months, much like influenza virus and RSV. This finding was also observed in other HMPV studies, though sampling only occurred during the winter months in those studies (*1*,*4*,*6*,*9*). The identification of HMPV-positive children in May and August, however, suggests that HMPV may continue to circulate in the spring, as was observed in a previous study (*13*), or possibly year-round.

In the United States, outbreaks of RSV disease tend to occur in the southern United States several weeks before the northeastern United States (*18*). In this study, HMPV infections were first identified in Rochester 7 weeks before the first infections in Nashville (data not shown). This finding may indicate that HMPV circulation does not exhibit the same pattern of activity as RSV, but our small number of HMPV-positive patients and 1 year of data limit our conclusions. Multiple years of surveillance, encompassing a wide geographic area, are needed to verify this preliminary observation.

Our study is subject to several limitations. The use of combined nose and throat swab specimens, as opposed to nasopharyngeal aspirate specimens, may have resulted in a substantial number of false-negative results. In particular, RSV detection seems to vary substantially by the type of specimen collected (*19*,*20*). Whether HMPV detection also varies by specimen type in a similar manner is unknown. Similarly, the use of frozen aliquots of specimens shipped in lysis buffer may have decreased virus yield somewhat for all the pathogens tested. We may have detected viral RNA from an earlier acute infection but not directly associated with the study illness. The amount and duration of shedding of HMPV has not been well described. Finally, the small number of positive HMPV patients limits our ability to fully characterize features of HMPV infection.

Nevertheless, our data suggest that HMPV infection is associated with severe respiratory illness in children. Further study is now needed to fully explore the link between disease and infection, assess seasonality patterns and incidence of disease, and examine risk factors for severe disease with infection.
